# The Association Between Father Support and Daughter Physical Activity: Insights From a Study Involving U.S. Latina Pre-Teens

**DOI:** 10.1177/08901171251315022

**Published:** 2025-01-18

**Authors:** Taynara Formagini, Alma I. Behar, Jennifer Schneider, Marisa Torres, Victoria M. Telles, Scott Roesch, Guadalupe X. Ayala, Tom Baranowski, Becky Marquez, Elva M. Arredondo

**Affiliations:** 1 Department of Family Medicine, 8784 University of California San Diego, La Jolla, CA, USA; 2 Institute for Behavioral and Community Health, 7117 San Diego State University, San Diego, CA, USA; 3 School of Public Health and the Institute for Behavioral and Community Health, 7117 San Diego State University, San Diego, CA, USA; 4 8784 Joint Doctoral Program in Public Health at San Diego State University-University of California San Diego, San Diego, CA, USA; 5 Pediatrics-Nutrition, 3983 Baylor College of Medicines, Houston, TX, USA; 6 The Herbert Wertheim School of Public Health and Human Longevity Science, 8784 University of California San Diego, La Jolla, CA, USA; 7 Department of Psychology, San Diego State University, San Diego, CA, USA

**Keywords:** moderate-to-vigorous physical activity, latina pre-teens, father support, body mass index, accelerometer-assessed physical activity

## Abstract

**Purpose:**

Social support, particularly from family, is crucial for physical activity (PA) among youth. This study examined the association between father support and moderate-to-vigorous physical activity (MVPA) in Latina pre-teens and explored the moderating role of body mass index (BMI).

**Design:**

Cross-sectional analysis.

**Setting:**

Baseline data from a pilot randomized controlled trial in San Diego County.

**Subjects:**

Sixty Latina pre-teen girls aged 8-11 years.

**Measures:**

Girls’ MVPA was measured via accelerometry. An exploratory factor analysis created a composite measure of father support, reported by mothers.

**Analysis:**

Hierarchical linear regression models, adjusting for covariates, assessed the relationship between father support and MVPA. Interaction models tested the moderating effect of BMI z-score (zBMI).

**Results:**

Father support was significantly associated with MVPA (b-range = 0.07 to 0.08, 95%CI = 0.02, 0.13) after adjusting for age, income, acculturation, and maternal support. However, this association became non-significant with the inclusion of zBMI (b = 0.06, 95%CI: −0.01, 0.11). The interaction model showed a significant positive association between father support and MVPA among girls with a healthy weight (b = 0.27, 95%CI: 0.27, 2.68) but not among those with overweight/obesity (b = 0.95, 95%CI: −0.38, 2.28).

**Conclusion:**

Our findings indicate a potential association between father support and daughters’ MVPA among girls with a healthy weight. Further research is needed to understand why this association is not observed in girls with overweight/obesity.

## Purpose

Physical activity (PA) significantly impacts children’s health and well-being and is associated with a lower risk for overweight/obesity, improved musculoskeletal health, enhanced academic performance, and increased social and emotional development.^1-4^ Engaging in PA during childhood yields lifelong health advantages, including the prevention of cardiovascular and chronic conditions in adulthood.^5-7^ Yet, only about 25% of U.S. children and adolescents meet PA guidelines of 60 or more minutes of daily moderate-to-vigorous PA (MVPA), with a noted decline in activity as adolescents transition into adulthood.^8,9^ Notably, fewer girls (18%) meet the PA guidelines compared to boys (35%), and children with overweight/obesity tend to engage in less PA than those with a healthy weight.^10,11^ Importantly, Latina pre-teen girls perform less PA compared to their male counterparts.^12-14^ Increasing MVPA among girls holds promise for shaping their overall PA habits into adolescence and adulthood, with the potential to reduce the risk of obesity and improve cardiovascular health in women.^15,16^

Strategies that harness family-level influences show potential for promoting PA among pre-teen girls, given the critical role parents play in shaping their children’s health behaviors.^17-19^ Parents can influence PA through social support, such as joint exercise (ie, co-participation, modeling), encouragement, providing transportation to PA, reinforcing children to be physically active, and offering information about PA benefits.^20-24^ The impact of social support might be even more substantial among Latinos due to *familismo*, a core value in Latino culture, in which strong family ties and perception of the importance of family as a source of support, strength, and inspiration are principles that guide behavior and decision-making.^25^ There is robust evidence among Latino families highlighting the positive impact of maternal support on daughters’ PA levels,^26-29^ but leveraging father support in interventions could also be a strategy to promote girls’ PA. Qualitative research indicates that Latino-specific cultural norms influence the dynamics of the father-daughter relationship. These norms establish fathers not solely as authoritative figures impacting their children’s health behaviors and choices, but also as role models.^30-32^ Family-based interventions may capitalize on these paternal roles and target specific areas such as encouraging joint PA participation, educating on healthy lifestyle choices, and fostering positive family dynamics prioritizing health and well-being. However, quantitative research evaluating the relationship between paternal support and Latina girls’ PA in the U.S. remains limited, highlighting the need for further investigation in this area.^33,34^

Our study bridges this gap by examining the association between father support for PA and accelerometer-assessed MVPA among Latina pre-teens (8-11 years old). Unlike self-reported measures of PA, which remain prevalent in the literature, accelerometer-assessed PA offers a more objective and accurate method of capturing PA levels.^35^ Previous research suggests that parental support for PA may vary depending on the child’s weight status, with children with overweight potentially receiving less support from their parents.^36-38^ Thus, we also explored whether daughters’ BMI moderates the relationship between father support and daughters’ MVPA. A deeper understanding of the relationship between father support for PA and PA among Latina pre-teens could inform the development of family-centered behavior-change interventions that specifically leverage father support to increase PA levels among Latina pre-teens.

## Methods

### Design

This cross-sectional study utilized baseline data from the *Conmigo* study (“With me,” in English), a 12-week pilot randomized controlled trial of an online intervention conducted in 2020-2022 which aimed to promote PA among pre-teen daughters and their mothers.^39^ Investigators partnered with elementary schools and various community organizations (eg, churches, community centers, food banks) across San Diego, in areas with high concentrations of Latinos, to recruit mother-daughter dyads interested in participating in the study. The intervention was originally designed for in-person delivery; however, due to COVID-19 pandemic restrictions, it was adapted for online implementation. All components of the intervention were subsequently conducted virtually, utilizing a video conferencing platform to maintain program integrity while ensuring participant safety and adherence to public health guidelines. Further details regarding the study rationale and design are available elsewhere.^39^

### Sample

A total of 79 mother-daughter dyads were enrolled and randomized to participate in the Conmigo intervention or waitlist control (delayed abbreviated intervention). Criteria for participation in Conmigo included: 1) a daughter aged 8-11 years and not meeting the Centers for Disease Control and Prevention (CDC)’s 2018 PA guidelines (60 minutes of MVPA/day),^40^ 2) the mother as the primary caregiver, 3) both self-identifying as Hispanic/Latina, 4) English or Spanish language dominant, and 5) having access to internet-enabled electronic devices at home. The study took place in San Diego County, a region with a large Hispanic/Latino population with diverse socioeconomic backgrounds. While the sample is not fully representative of the broader Latino population in the U.S., participants’ sociodemographic characteristics (see Table 1) generally align with those of Latino families in urban settings across San Diego County.^41^ The study received approval from the Institutional Review Board of the San Diego State University [HS-2019-0197]. The present study utilized baseline data from a subset of daughters who had a father figure residing with them (as reported by mothers) and had completed accelerometer PA measurements at baseline (N = 60).

**Table 1.** Descriptive Characteristics of Dyads (N = 60).

Characteristic	Mean (SD) [range] or n (%)
*Covariates*	
Daughter’s age, mean (SD) [range]	9.8 (1.2) [8-11]
Family monthly household income, n (%)	
< $2000	11 (22.5)
$2001-$3000	13 (26.5)
≥ $3001	24 (49.0)
Prefer not to answer	1 (2.0)
Daughter’s acculturation score, mean (SD) [range]^a^	3.1 (0.6) [1.2-4.3]
Mother support for PA, mean (SD) [range]^b^	2.2 (1.0) [0.2-4.0]
Daughters’ zBMI categories, n (%)^c^	
Healthy weight	26 (53.1)
Overweight/obesity	23 (46.9)
*Other characteristics*	
Daughters’ nativity, n (%)	
U.S.	55 (93.2)
Mexico or other	4 (6.8)
Daughters’ ethnicity, n (%)	
Mexican/Mexican-American	57 (95.0)
Other Latin American background	3 (5.0)
Daughters’ language preference, n (%)	
English	43 (71.7)
Spanish	17 (28.3)
*Independent variable*	
Father support for PA, mean (SD) [range]^d^	2.3 (0.8) [0-4.0]
*Outcome variable*	
Daughters’ MVPA (minutes/day), mean (SD) [range]^e^	23.3 (12.9) [0.5-58.2]

Where the total does not add to 60, participants were missing data on that characteristic.

^a^The Short Acculturation Scale for Hispanic Youth (SASH-Y) was calculated using an average rating across all 12 items. Scores above 2.9 represent high acculturation.

^b^Calculated by deriving a composite score from two sub-scales of the Physical Activity Parenting Practices (PAPP) on a five-point scale, ranging from 0 (never) to 4 (very often), with higher scores indicating greater levels of support.

^c^BMI was calculated from mother-reported height and weight using the CDC age- and sex-specific BMI percentile to generate weight categories, defined as healthy weight (<85th percentile) and overweight/obesity (≥85th percentile).

^d^Ranges from zero to 4, where higher scores indicate higher support.

^e^Measured using Actigraph devices (GT3X+ and wGT3x-BT) worn for 3-7 days for ≥10 hours/day.

### Measures

The Conmigo study collected data at baseline from mothers and daughters. Daughters completed surveys via phone with a bilingual/bicultural research assistant. Mothers self-administered surveys and completed questions about father support for PA using a Qualtrics link.^42^

#### Independent Variable

Father support for daughters’ PA was measured using a 5-item scale adapted from Sallis et al. (2002).^43^ Mothers reported how often the father or father-figure in the household would 1) encourage their daughter to do PA or play sports (emotional support); (2) play outside or engage in PA or sports with their daughter (companionship); (3) provide transportation to a place where their daughter can engage in PA or play sports (instrumental support); (4) watch their daughter participate in sports, PA, or outdoor games (validation); and (5) tell their daughter that PA is good for her health (informational support). Responses were recorded on a five-point scale with endpoints ranging from zero (never) to 4 (daily), where higher scores indicate higher total provision of social support. In the current sample, the internal consistency of the scale was α = 0.82.

#### Outcome Variable

Average minutes/day of MVPA were measured via Actigraph devices (GT3X + or wGT3x-BT). These devices were initialized in Actilife Software (version 6.13.4) to record raw acceleration at 30 Hz. Following initialization, the research team mailed the devices to participants along with written and video instructions detailing proper wearing techniques (ie, positioning the device on the right hip, above the iliac crest, using a fitted belt). Daughters were asked to wear the device during all waking hours and to remove it during water-based activities (eg, shower or pool) and while sleeping. Research staff conducted follow-up calls after mailing the devices to address any questions. After 7 days, mothers either returned the device via mail or had it picked up by the research team at their homes. Raw acceleration was subsequently converted into 60-second epochs for analysis using counts per minute (CPM) cut-points, with MVPA defined as at least >2295 CPM.^44^ Valid accelerometer wear was defined as wearing the device for at least 3 days with a minimum of 10 hours/day.^45^ Daughters with invalid accelerometer wear data were asked to re-wear the devices for a second 7-day period.

#### Moderator Variable

Daughter’s BMI z-score (zBMI) was calculated from mother-reported height and weight using the CDC’s age- and sex-specific BMI percentiles to generate weight categories, defined as healthy weight (<85^th^ percentile) and at-risk for overweight/obesity (≥85^th^ percentile).^46^

#### Covariates

We included 5 covariates in the analysis due to their potential association with parental support and PA among children: daughter’s age and acculturation level, family’s monthly household income, mother’s support for PA, and BMI.^47^ Age was self-reported by daughters in years. Daughters self-reported their adherence to cultural practices using the 12-item Short Acculturation Scale for Hispanics, Youth Version (SASH-Y).^48^ An acculturation score is calculated by obtaining an average rating across all 12 items; nine items represent language use (English vs Spanish) and 3 items represent social interactions (Hispanic/Latino vs non-Hispanic/Latino). Response options range from 1 = Only Spanish to 5 = Only English. A score of 2.9 represents the cut-point for high acculturation, greater assimilation to the Anglo culture as represented by more English language use, and association with non-Hispanics/Latinos. Monthly household income was reported by mothers using a categorical variable and then recoded into 3 levels, where 1: <$2000/month, 2: $2001-$3,000, and 3:≥ $3001/month. Mother support for PA was calculated by deriving a composite score from 2 sub-scales of the Physical Activity Parenting Practices (PAPP), specifically nondirective support and autonomy support, comprising 5 total questions.^49^ Daughters reported their mothers’ support levels responding to a five-point scale that ranged from zero (never) to 4 (very often), with higher scores indicating greater levels of support.

### Analysis

Daughters’ characteristics were summarized using frequencies for categorical variables and mean and standard deviations for continuous variables. An exploratory factor analysis using maximum likelihood estimation informed a composite measure of father support of daughter’s PA. This process assigns appropriate weights to each variable in the scale, facilitating a comprehensive understanding of the underlying constructs.^50^

Linear regression models, employing a hierarchical approach adjusting for covariates in subsequent models, were used to assess the association between father support and daughters’ MVPA. We also conducted separate interaction models to examine whether daughters’ zBMI moderated the association between father support and daughters’ MVPA, stratifying by zBMI (ie, healthy weight vs overweight/obesity) (n = 49). A separate category was created for missing data on covariates to retain all individuals in the analysis and ensure comparability across models. This approach minimizes data loss and maintains sample size, which is relevant given our sample size. Participants with missing BMI data (n = 11) were less acculturated (eg, greater likelihood of using Spanish language), had similar household incomes, similar mother support for PA, and lower levels of MVPA compared to those with complete BMI data. All analyses were performed using STATA SE software version 18,^51^ and statistical significance was set at *P* < 0.05. The coding scripts can be found on GitHub (www.github.com/taynaraformagini/Conmigo_father_support).

## Results

### Participant Characteristics

The mean age of daughter participants was 9.8 years old (SD = 1.2), and almost half of the participants lived in a household with a monthly income of over $3001 (49%). Most daughters were born in the U.S. (93%) and identified as Mexican/Mexican-American (95%). Most daughters reported that English was their preferred language (72%). Acculturation levels were high within our sample, as reflected by a mean acculturation score of 3.1 (on a 5-point scale), which exceeded the high acculturation threshold of 2.9 by 0.2 points. The average level of mother support for PA among participants was 2.2 on a scale ranging from zero to 4, indicating that the support was slightly above average. Based on accelerometer data, daughters engaged in an average of 23 minutes/day of MVPA. Over half of the daughter participants (53%) were classified as having a healthy weight, while 47% were classified as having overweight/obesity. (Table 1).

### Father Support and PA

Scores for father support for PA ranged from zero to 4, with a mean score of 2.3, indicating slightly above-average support from fathers among the participants (Table 1).

### Total Father Support for Daughter PA Using Factor Analysis

Exploratory factor analysis revealed a clear factor structure, with Factor 1 showcasing the highest eigenvalue of 2.45738. This value, surpassing the threshold of 1 that indicates a significant variance explanation, underscores the primary importance of Factor 1 in elucidating the scale’s variance (Table 2).

**Table 2.** Factor Analysis Using Maximum Likelihood Estimation to Estimate Total Father Support for Daughters’ Physical Activity.

Factor	Eigenvalue	Proportion
1	2.45738	0.9701
2	0.40766	0.1609
3	0.00359	0.0014
4	−0.08973	−0.0354
5	−0.24571	−0.0970

Table 3 presents the Factor 1 loadings for each variable of the father support for PA scale. Factor loadings indicate the strength and direction of the relationship between each variable and the underlying factor (father support for PA). Higher factor loadings, such as those seen for “Encourage daughter to do PA or play sports” and “Play outside or do PA or sports with daughter,” signify stronger positive relationships with Factor 1. Conversely, lower factor loadings, such as that for “Tell their daughter that PA is good for her health,” indicate weaker relationships. These five-factor loadings were used to calculate an aggregate measure of father support for PA by weighting each variable based on its contribution to Factor 1.

**Table 3.** Factor 1 Loadings With the Weight of Each Variable of the Father Support for Physical Activity Scale.

Variable	Factor 1 loadings
Encourage daughter to do PA or play sports	0.7627
Play outside or do PA or sports with daughter	0.7186
Provide transportation to a place daughter can do PA or play sports	0.5204
Watch their daughter participate in sport, PA or outdoor games	0.2975
Tell their daughter that PA is good for her health’	0.2114

### Associations Between Father Support for PA and Daughter MVPA

Table 4 describes the associations between father support for PA and daughters’ MVPA (mean minutes/day), utilizing a hierarchical approach to adjust for covariates in subsequent models. A significant positive association between father support and daughter MVPA emerged in the unadjusted model (*b* = 0.08, 95%CI: 0.02.0.13), and remained significant in models adjusted for age, household income, daughter acculturation, and mother support (*b* range: 0.07, 95%CI: 0.01, 0.13). However, after further adjustment for the daughter’s zBMI, the association became non-significant (*b* = 0.06, 95%CI: −0.01, 0.11). In light of this, we explored the impact of daughters’ zBMI as a moderating variable in the relationship between father support and daughters’ MVPA.

**Table 4.** Linear Regression Models of the Association Between Father Support for PA and Daughters’ MVPA (n = 60).

	*b*	r-squared	95% CI
**Unadjusted**	0.08	0.12	0.02,0.13*
**+ Daughter age**	0.07	0.13	0.02,0.13*
**+ Household income**	0.07	0.19	0.02,0.13*
**+ Daughter acculturation**	0.07	0.20	0.02,0.13*
**+ Mother support for PA**	0.07	0.22	0.02,0.12*
**+ Daughter zBMI**	0.06	0.26	−0.01,0.11

Abbreviations: β (95% CI) = Coefficient; 95% CI = 95% confidence interval; MVPA = Moderate to vigorous physical activity; PA = Physical activity. *statistically significant at *P*-value <0.05.

### The Moderating Role of Daughter’s zBMI in the Association Between Father’s Support for PA and Daughter MVPA

We conducted linear regression models examining daughters’ zBMI as a moderator in the relationship between total father support for PA and daughters MVPA, adjusting for daughters’ age and acculturation, household income, and mother support for PA (Table 5). Among daughters’ with a healthy weight (n = 26), the association between girls’ zBMI-father support interaction and MVPA revealed a significant positive association in the unadjusted model (*b* = 1.48, 95%CI: 0.27, 2.68) and in the models adjusting for daughter age and acculturation, and household income (*b range* = 1.40 to 1.50, 95%CI: 0.27, 2.68). However, among daughters with overweight/obesity (n = 23), the zBMI-father support interaction was not significantly associated with MVPA across any of the models.

**Table 5.** Linear Regression Models of the Moderator Effect of Daughter’s BMI on Father Support for PA and Daughters’ MVPA (n = 49).

	Healthy weight (n = 26)		Overweight/obesity (n = 23)		
	*b*	95% CI	*b*	95% CI	r-squared
**Unadjusted**	1.48	0.27,2.68*	0.95	−0.38,2.28	0.13
**+ Daughter age**	1.40	0.18,2.61*	0.77	−0.62,2.16	0.14
**+ Household income**	1.50	0.16,2.83*	0.86	−0.16,2.83	0.16
**+ Daughter acculturation**	1.50	0.15,2.85*	0.85	−0.68,2.39	0.16
**+ Mother support**	1.21	−0.13,2.56	0.50	−1.03,2.03	0.24

Abbreviations: β (95% CI) = Standardized coefficient; 95% CI = 95% confidence interval; MVPA = Moderate to vigorous physical activity. *statistically significant at *P*-value <0.05. Note: 49 participants were included in the analysis because 11 participants had missing BMI data.

## Discussion

Our study examined the association between father support for PA and daughters’ MVPA among a sample of 60 pre-teen Latina girls in San Diego County. To our knowledge, this is the first study to assess whether father support for PA is associated with objectively measured MVPA among Latina pre-teens in the U.S. Our exploratory findings indicate a potential relationship between father support and daughters’ MVPA. We also observed a positive trend between father support and MVPA among daughters with a healthy weight. Interestingly, these findings did not hold for daughters classified as having overweight/obesity. Given that Latina girls engage in insufficient PA, data that provide potential mechanisms of change that can be intervened upon are valuable to inform targeted interventions aimed at promoting PA and improving health outcomes in this group. These preliminary findings suggest a potential avenue for further exploration into how father support plays a role in the PA levels of Latina girls, and Latino children more broadly.

The association between father support for PA and Latina daughters’ MVPA is consistent with prior research demonstrating that parental support for PA positively influences children’s PA across diverse ethnic groups in the U.S. and globally. For instance, a comprehensive study in China involving over 61 000 school-aged children (6-18 years) demonstrated that various forms of parental support were positively associated with increased child MVPA.^52^ Similarly, cross-sectional studies in Japan and the United Kingdom identified a link between parental support for PA and MVPA in children aged 8-9 years.^53,54^ Extending these international findings, multiple studies within the U.S. have consistently pointed to the potential impact of family support on children’s PA levels.^47,55-58^ Notably, a meta-analysis assessing the impact of parental modeling and support behaviors on child PA highlighted a correlation between father-daughter PA, comparable in strength to mother-daughter PA.^59^ Although research among Latino populations is scarce, research involving other populations has substantiated a positive association between father support and daughter PA. For example, a study in Denmark found that various forms of father support (eg, encouragement, participation, observation, and discussion about PA) were associated with MVPA among adolescent girls (ages 11-15 years).^60^ Yet, prior interventions have predominantly focused on increasing maternal support for daughters’ PA, and only a few have been successful in increasing PA among fathers and daughters.^61,62^ 1 ongoing trial of a father-focused lifestyle program targeting obesity-related behaviors (eg, PA, nutrition) among low-income Latino men has been shown to be feasible, and participants reported improved father-child bonding.^63,64^ Our study adds to this evolving area of research by highlighting the potential role that fathers play in supporting their Latina pre-teen daughters’ engagement in MVPA and emphasizes the need to further explore the nuanced dynamics of Latino father-daughter relationships and their impact on daughters’ PA behaviors.

It is well-documented that children’s demographic, psychological, behavioral, and physical characteristics are correlated with their PA levels.^47^ Notably, children’s BMI has been inversely associated with PA, especially among girls.^65^ Our exploratory analyses indicate that daughter’s weight status might play a moderating role in the relationship between father support for PA and daughter’s PA. Prior research indicates that children with overweight/obesity may encounter more barriers to PA compared to children with a healthy weight.^36,66,67^ Particularly, girls with overweight or obesity report receiving less support for PA from their parents compared to girls with a healthy weight.^36-38^ Consistent with these findings, our study observed that father support was significantly associated with their daughter’s measured MVPA only among those with a healthy weight (measured by zBMI). This may suggest that the relationship between father support and PA may be influenced by the daughter’s weight status, thus raising concerns about the nature of parental support for PA among girls with overweight/obesity. For instance, children whose parents believe they are competent in relation to physical fitness tended to be more physically active compared to children whose parents hold lower perceptions of their fitness competence.^68^ Furthermore, research among Latinos has linked paternal machismo with unsupportive behaviors toward their children, prompting consideration of its potential impact on support provided to girls with overweight/obesity.^69^ Alternatively, these findings could indicate that the barriers to PA faced by girls with overweight/obesity, such as societal stigma or physiological challenges, may diminish the impact of parental support on their activity levels. Future research should explore the specific types of support that are most effective in promoting PA among girls with a healthy weight and girls with overweight/obesity, as well as identify potential barriers that hinder parental support among girls with overweight/obesity.

Moreover, this study was conducted during the COVID-19 pandemic, a period that may have influenced levels of father support for daughters’ PA as well as overall PA levels. The pandemic disrupted daily routines, creating both barriers and opportunities for social support and PA.^70-72^ Some studies have reported decreased PA levels and social support during the pandemic due to factors such as restricted access to outdoor spaces and increased caregiving burdens,^73-76^ while others have observed increased family bonding time and support for PA as families engaged in more home-based activities.^77-80^ This mixed evidence suggests that the pandemic’s effect on father support for daughters’ PA could vary, making it unclear in which direction it may have impacted our findings. Although our study collected data during the pandemic, the findings remain valuable for understanding the mechanisms of father support for daughters’ PA, as family dynamics and support for PA play a key role in both pre- and post-pandemic contexts. Future research could examine whether the patterns we identified hold in a post-COVID context to better understand the stability of these associations over time.

Our study has several limitations. First, the small sample size may have limited our ability to detect significant associations or have confidence in the relationships detected, highlighting the need for caution when interpreting the results. Similarly, the sample may not be representative of the broader Latino population, as participants were drawn from a single geographic region in California, which may have unique cultural and environmental factors. Second, the cross-sectional study design restricts inference to associations only and does not allow for establishing causality or temporal relationships. Third, relying on mothers’ reports of fathers’ social support (versus father-report or daughter-report on father) and on their daughter’s height and weight may have introduced potential biases. Regarding the former, although previous research using the same support scale reported parent and child reports correlated significantly (r = 0.61, *P* < 0.01),^45^ perceptions of support can vary among girls compared to their mothers’ perceptions. Regarding the latter, the constraints imposed by the COVID-19 pandemic restricted our ability to collect objective measures for height and weight, necessitating reliance on self-reports. Lastly, while the scale assesses multiple types of support (ie, instrumental, emotional, companionship, validation, and informational), and we have adjusted the models for mother support, it does not account for support from other influential figures, such as siblings or peers, who may also impact teens’ PA. In spite of these limitations, the use of accelerometer-assessed PA data adds strength to the study findings. Accelerometry is a valid and reliable tool to objectively assess PA. This method stands superior to self-reported measures of PA, which remain prevalent in the literature.^35^

More research is needed to understand the role of father support on daughter’s PA among Latino families. Future studies with larger sample sizes and longitudinal designs are warranted to provide a more comprehensive understanding of these associations. Exploring different dimensions of father support (eg, informational, emotional, instrumental) may also provide an understanding of the specific types of support that are most influential in promoting PA among pre-teen Latinas. Research is also needed among a larger age range of Latina girls to determine if the observed associations between father support and daughter MVPA are consistent across various developmental stages. Examining other potential moderators and mediators in this association is also crucial. For instance, investigating whether differences exist in father support among sub-groups of Latinas based on factors such as country of origin or acculturation levels could provide valuable insights into the nuanced dynamics at play. Assessing how daughter support may, in turn, enhance the father’s PA levels could also offer a more comprehensive understanding of reciprocal support within families.

### Conclusions

This study contributes to the limited research on the association between paternal support and child PA among Latino families by shedding light on the complex interplay among father support, daughter PA, and daughter weight status among pre-teen Latinas. While we found significant associations between father support for PA and daughter MVPA among girls with a healthy weight, the lack of significant associations among girls with overweight/obesity suggests potential disparities in paternal support for PA based on daughter’s weight status. Additional research is needed to further examine the nuances of father-daughter relationships, consider longitudinal designs, and assess the effectiveness of family-based interventions targeting fathers in promoting healthy lifestyles among Latina youth.

**So what?**

**What is Already Known on This Topic?**

Family support plays a key role in promoting physical activity (PA) among youth. However, the specific impact of father support on the PA of Latina pre-teens remains underexplored.

**What Does This Article Add?**

This study suggests a potential positive association between father support and moderate-to-vigorous physical activity (MVPA) in Latina pre-teens, particularly among those with a healthy weight. The findings indicate that this relationship may vary based on the child’s weight status.

**What are the Implications for Health Promotion Practice or Research?**

These results highlight the need for further research to better understand the role of father support in promoting PA among Latina pre-teens, especially considering how factors such as weight status may influence this relationship. Future studies should explore these dynamics in larger, more diverse samples to inform family-based interventions.
